# Health Effects and Public Health Concerns of Energy Drink Consumption in the United States: A Mini-Review

**DOI:** 10.3389/fpubh.2017.00225

**Published:** 2017-08-31

**Authors:** Laila Al-Shaar, Kelsey Vercammen, Chang Lu, Scott Richardson, Martha Tamez, Josiemer Mattei

**Affiliations:** ^1^Department of Nutrition, Harvard T.H. Chan School of Public Health, Boston, MA, United States; ^2^Population Health Sciences Program, Graduate School of Arts and Sciences, Harvard University, Cambridge, MA, United States; ^3^Department of Epidemiology, Harvard T.H. Chan School of Public Health, Boston, MA, United States

**Keywords:** energy drinks, beverages, review, health effects, food policy, regulation

## Abstract

As energy drink consumption continues to grow worldwide and within the United States, it is important to critically examine the nutritional content and effects on population health of these beverages. This mini-review summarizes the current scientific evidence on health consequences from energy drink consumption, presents relevant public health challenges, and proposes recommendations to mitigate these issues. Emerging evidence has linked energy drink consumption with a number of negative health consequences such as risk-seeking behaviors, poor mental health, adverse cardiovascular effects, and metabolic, renal, or dental conditions. Despite the consistency in evidence, most studies are of cross-sectional design or focus almost exclusively on the effect of caffeine and sugar, failing to address potentially harmful effects of other ingredients. The negative health effects associated with energy drinks (ED) are compounded by a lack of regulatory oversight and aggressive marketing by the industry toward adolescents. Moreover, the rising trend of mixing ED with alcohol presents a new challenge that researchers and public health practitioners must address further. To curb this growing public health issue, policy makers should consider creating a separate regulatory category for ED, setting an evidence-based upper limit on caffeine, restricting sales of ED, and regulating existing ED marketing strategies, especially among children and adolescents.

## Introduction

Energy drinks (ED) are non-alcoholic beverages marketed to improve energy, stamina, athletic performance, and concentration. Categorized as “functional beverages” alongside sports drinks and nutraceuticals, the ED industry has grown dramatically in the past 20 years, reaching over $9.7 billion in United States (U.S.) sales in 2015, with two brands accounting for nearly 85% of the market (1). The target consumer market for ED is adolescents and young adults (1), with one study finding that 51% of college students report consuming at least one ED each month (2). While annual sales of ED remain dwarfed by those of soft drinks and coffee, there are concerns that lax regulation of marketing and ingredient labeling is spurring a trend of increased consumption. Since their introduction to the U.S. market in 1997, there has been a significant increasing trend in ED consumption among children, adolescents, and adults, although the proportion of calories attributable to ED is still marginal compared to other sugar-sweetened beverages (SSBs) such as soda and fruit juice (3, 4). The rising prevalence of ED consumption is particularly problematic given the emerging evidence of association with negative health consequences such as risk-seeking behaviors, adverse cardiovascular effects, and metabolic, renal, or dental conditions (5, 6). Updating and integrating the findings of some previous reviews (7, 8), this mini-review summarizes the current scientific evidence on ED health effects, presents relevant public health challenges, and proposes recommendations to amend these issues.

## Constituents and Ingredients

Despite the vast array of ED available in the U.S., most ED contain similar ingredients including water, sugar, caffeine, non-nutritive stimulants (e.g., guarana, ginseng, yerba mate, taurine, l-carnitine, d-glucuronolactone, and inositol) and certain vitamins and minerals (e.g., B vitamins) (1, 9). The caffeine content in ED ranges widely from 47 to 80 mg per 8 oz to as high as 207 mg per 2 oz and comes from a number of ingredient sources (10). While moderate caffeine intake (up to 400 mg/day) is generally considered safe and even beneficial for health among adults (11), there has not been extensive research conducted on children and adolescents to determine if any tolerable level exists (12). ED also contain large amounts of high fructose-corn syrup, sucrose, or artificial sweeteners. The amount of sugar contained in one can (500 mL or 16.9 oz) of an ED is typically about 54 g (13). Many institutions, including the World Health Organization, have recommended reducing sugar intake due to the strong evidence linking consumption of added sugar to poor health (14).

Reports on other constituents of ED are relatively limited. Guarana is a plant extract native to South America which contains a significant amount of caffeine, with 1 g of guarana equivalent to 40 mg of caffeine (15). Due to this particularly high caffeine content, guarana is often included as an ingredient in ED for its stimulatory effect (1, 16). Ginseng is an herbal supplement that has been used for thousands of years in East Asia and has reported health benefits including vasorelaxation, antioxidation, anti-inflammation, and anticancer (17, 18). Like guarana, yerba mate has a high caffeine concentration (78 mg caffeine per cup) (1) and is additionally thought to have benefits in the form of antioxidant capacity, weight management, and cancer prevention (19). Taurine has been reported to have anti-inflammatory actions and has been suggested in the treatment of epilepsy, heart failure, cystic fibrosis, and diabetes (20). B vitamins refer to a group of eight water soluble vitamins which generally play an important role in cell functioning, with vitamin B2 (riboflavin), B3 (niacin), B6 (pyridoxine, pyridoxal, and pyridoxamine), and B12 being the most common B vitamins added to ED (1). Despite the importance of B vitamins as coenzymes in various metabolic processes, most individuals in the U.S. already meet the recommended daily amount and hence the additional B vitamins added to ED are often excreted from the body with urine, failing to impart any additional health effect (1). The literature on the content and function of other additives such as l-carnitine, d-glucuronolactone, and inositol is limited, with a few studies suggesting moderate benefits (18).

## Health Effects of ED

### Improved Cognitive and Physical Performance

Some studies support the temporary health benefits of ED in improving mental and physical stamina among both adults and adolescents. Several randomized controlled trials among adults have shown an association between components of ED and improved subjective alertness (21), as well as restoration of fatigue (22). Due to its similar structure to adenosine, caffeine can inhibit sleep through its competitive binding to the adenosine receptor (23). Studies have also shown the effect of ED on improved physical activity performance in young-adult athletes (24–27). A recent meta-analysis of acute-effect studies conducted among adults found that ED consumption improved muscle strength and endurance, performance on endurance exercise tests, jumping, and sport-specific actions (28).

In contrast, the vast majority of evidence suggests negative health effects of both short- and long-term ED consumption (Table 1), with most literature proposing these health disadvantages are attributable to high levels of caffeine and sugar while highlighting that more research is needed on the effects of other ED constituents.

**Table 1:** Summary of negative health effects of energy drinks (ED).

Category of health effects	Major effects observed
Risk-seeking behaviors	Substance abuse: alcohol, cigarettes, marijuana, amphetamines (32, 35) Aggressive behavior: fighting, bullying, truancy (35)
Mental health effects	Stress, anxiety, depressive symptoms, suicidal ideation, plan or attempt (33, 36, 37) Low academic achievement (35)
Adverse cardiovascular effects	Increased systolic and diastolic blood pressure (39, 41) Increased heart rate (39)
Adverse metabolic, dental, or renal effects^a^	Overweight/obesity risk (44, 47–49) Risk of metabolic/type 2 diabetes (48) Dental decay (51, 52) Renal microvascular damage and accelerated progression of chronic kidney disease (53–55)
Other health effects	Sleep dissatisfaction, tiredness/fatigue, late bedtime, headaches, stomachaches and irritation (35, 56–59)

*^a^The evidence for adverse metabolic and dental effects comes from studies on sugar-sweetened beverages in general; ED are often classified as such as they contain added sugar*.

### Risk-Seeking Behaviors and Mental Health Effects

Several studies report a consistent association between ED consumption and substance abuse, although the evidence base consists primarily of cross-sectional studies, which does not allow for establishing directionality of the association (29–34). For example, in a nationally representative sample of U.S. middle- and high-school students, significant associations were found between ED frequency and 30-day frequency of use of alcohol, cigarettes, marijuana, or amphetamines (32). This echoes cross-sectional results among adolescent regular consumers (>1 ED/week) in Europe who were more likely to smoke and drink alcohol compared to non-consumers (35). Some studies have additionally found evidence for an association between ED consumption and mental health, including stress, anxiety, depressive symptoms, and suicidal ideation, plan or attempt (33, 36, 37). For example, teenagers in Canada who reported ED consumption more than once a month were nearly three times more likely to report elevated depressive symptoms compared to those who did not report ED consumption (33). A review of ED consumption and mental health among adolescents and adults supported a positive association between chronic ED use and undesirable mental health effects, including stress, anxiety, and depression (37). The authors postulate that this association may be moderated by dysregulated sleep, such that consuming heavily caffeinated ED may result in sleep loss which in turn may contribute to poor functioning and mental health.

### Adverse Cardiovascular Effects

Numerous studies have explored the short-term effects of ED on the cardiovascular system, primarily with respect to caffeine and sugar (38–40). For example, a recent randomized crossover study on healthy subjects found that consumption of 355 mL of an ED resulted in increased systolic and diastolic blood pressure, heart rate, and cardiac output (39). A 2016 meta-analysis of 15 studies similarly reported that acute ED consumption resulted in increased systolic and diastolic blood pressure across the pooled results (41). Although the meta-analysis did not find evidence for increased heart rate, the researchers noted the need for well-designed studies before any conclusions can be made.

Caffeine toxicity is believed to occur above 400 mg/day for adults, 100 mg/day for adolescents (12–18 years), and 2.5 mg/kg of body weight for children (<12 years), with serious symptoms often related to cardiovascular effects (42). In the 1-year period between October 2010 and September 2011, the U.S. National Poison Data System received 4,854 ED-related calls, including major adverse events such as seizure, dysrhythmia, and tachypnea (42). Data from Australian poisons centers confirm these major symptoms of recreational or accidental ED intake among children and adolescents, in addition to palpitations, agitation, and tremor (43). Given that these data relies on self-reported signs and symptoms and most consumers may not readily identify ED as a poison, it is likely that ED-related toxicity reports are underestimated. It is believed that the potential for caffeine toxicity from ED is greater than other caffeine sources such as coffee or tea due to inadequate labeling and greater volume of consumption driven by heavy advertising promoting “more is better,” especially among children and youth (5).

### Adverse Metabolic, Dental, or Renal Effects

Sugar-sweetened beverages, which refer to beverages with added sugar such as soda, fruit juice, and many ED, are consistently associated with long-term negative health effects particularly among children and adolescents (44–46). Primarily, SSBs have been linked to overweight/obesity risk and metabolic conditions such as type 2 diabetes (44, 47–49), potentially through the low satiety of SSBs and consumers not sufficiently reducing total energy intake to account for the additional calories of SSBs (50). In addition, consumption of SSBs increases blood glucose and insulin levels, contributing to a high glycemic load which is associated with glucose intolerance and insulin resistance (48). SSBs are also associated with a high prevalence of dental caries, wherein bacteria in the mouth utilize the sugars from SSBs and produce acid that decays the teeth (51, 52). Finally, renal diseases, specifically renal microvascular damage (53) and accelerated progression of chronic kidney disease, have been shown to be induced by fructose in SSBs in animal-based models (54, 55).

### Other Health Effects

Energy drinks’ consumption is also associated with other commonly reported health problems such as sleep dissatisfaction, tiredness/fatigue, late bedtime, headaches, and stomachaches and irritation (35, 56–59). It is likely that many of these general health complaints are attributable to caffeine or sugar content, but additional research needs to be conducted to confirm this, as well as to assess the potential effect of other constituents. In a cross-sectional study conducted in Finland, consumers of ED had a 4.6 (95% CI: 2.8, 7.7) times greater odds of headaches, 3.6 (95% CI: 2.2, 5.8) times greater odds of sleeping problems, and 4.1 (95% CI: 2.7, 6.1) times greater odds of having an irritable mood compared to non-consumers (57).

### Limitations of the Existing Literature

The literature generally suggests that the negative health effects of ED outweigh the beneficial effects. However, there are considerable limitations to the existing literature. First, most studies on ED are cross-sectional, limiting the ability to establish temporal relationships and support causality. In addition, many articles used small homogenous populations, often consisting of healthy, young to middle-aged adults, thus limiting generalizability to the U.S. population. Finally, studies specifically examining the effects of other ED constituents are lacking and thus there is limited knowledge on the potential mechanisms beyond those of caffeine and sugar, for which most of the literature exist.

## Relevant Public Health Challenges

### Energy Drink Marketing

Since 2004, aggressive advertising by ED companies has led to substantial market growth, with a more than 240% increase in sales in the U.S. and worldwide. ED are now available in more than 140 countries (8) and are on track to become a $21 billion industry in the U.S. by 2017 (60).

Energy drinks’ marketing strategies pose a significant public health threat in the U.S. due to the size of their target adolescent market and the marketing strategies they employ. ED face heavy competition for market share with SSB corporations that dwarf them in size, customer base, distribution capability, and ability to invest in advertising (61). To compete, ED companies target the approximately 33.5 million 12- to 19-year olds in the U.S. (62). The public health implications of targeting adolescents are not trivial: adolescents lack maturity in key areas of the brain, are biologically predisposed to have poor impulse control, and are more likely to engage in risk-taking behavior (63). This not only makes them more likely to consume the product on a regular basis but also makes them more vulnerable to identifying and potentially engaging in sexual and risk-taking behaviors depicted in ED marketing.

Adolescents’ vulnerability to the marketing tactics of ED companies is particularly serious given that ED are now omnipresent across multiple vending channels with price points widely affordable to all strata of the population. There are few signs that the scale and targeting of ED marketing will change in the near future. A 2014 study on ED marketing conducted by the offices of several U.S. Senators found that of the sixteen companies they approached, only four agreed to avoid marketing their products to children (64).

### Lack of Regulation and Taxation

Overall, there is a significant lack of ED regulation in the U.S. (65). While the Food and Drug Administration (FDA) enforces a caffeine limit of 71 mg per 12 fluid ounces for soda, ED manufacturers can avoid this by classifying their product as a “supplement,” regulated under the 1994 Dietary Supplement and Education Act and not subject to any caffeine limits (1, 8). That said, many ED companies are moving to marketing their products as beverages to address public concerns that their products are circumventing labeling requirements and also to enable ED to be available to participants of the Supplemental Nutrition Assistance Program, a U.S. nutrition assistance program providing low-income individuals with financial support to purchase food and beverages.

In 2014, the American Beverage Association published the “Guidance for the Responsible Labeling and Marketing of Energy Drinks,” which allowed ED companies to voluntarily commit to a number of industry goals (66). The companies committed to report total quantities of caffeine from all sources, restrict marketing to children, and voluntarily report adverse events to the FDA. However, an assessment report found that compliance to this commitment was staggeringly low (64), with 8 of 12 companies still marketing to children and 4 of 10 companies not willing to report adverse events voluntarily. Other countries facing similar challenge to the U.S. have implemented various approaches to regulate ED. For example, Australia and New Zealand created a unique regulatory category for ED called “formulated caffeine beverages,” and set the upper limit of caffeine from any source to be 320 mg/L (65).

In addition to regulation, some countries have implemented taxation policies as a deterrent to ED consumption. Mexico currently taxes all non-alcoholic beverages with added sugar, including ED, at one-peso-per-liter, with recent assessments reporting that purchases of taxed beverages have decreased by an average of 6% since implementation in January 2014 (67). Similarly successful initiatives have been emerging in some U.S. cities (68).

### Alcohol and Energy Drink Mixing

In addition to being consumed alone, ED are frequently mixed with alcohol, with one study in the European Union finding that 71% of young adults report consuming ED with alcohol (69). This is problematic because individuals who drink alcohol-mixed ED consume more alcohol than if they were drinking alcohol alone (70, 71). Researchers attribute this to the fact that consumption of ED masks the signs of alcohol inebriation, enabling an individual to believe they can still safely consume more alcohol, leading to “awake drunkenness” (72). As a result of this increased alcohol consumption, those who drink alcohol-mixed ED are more likely to experience severe dehydration and alcohol poisoning (73). This negative health trend is particularly concerning as it disproportionately affects underage individuals and has been linked to binge drinking, alcohol-dependence behaviors (70) and drunk driving (74).

## Recommendations and Future Directions

Public health and policy action must be taken to mitigate the negative health effects and public health challenges associated with ED. First, the FDA should consider regulation of ED as a separate category, requiring clear labeling of total caffeine and sugar content in reference to daily recommended amounts, and enforcing an upper limit for caffeine based on current evidence. Additional consideration should be given to taxing ED and/or restricting the sale of ED to children and adolescents. Marketing strategies should be also regulated to minimize the promotion of ED among adolescent and young adults. Because marketing is largely aimed at this segment of the population, exposure to ED products could be reduced considerably. In parallel, further research should continue to improve the quality of the evidence on the health effects of ED with particular attention to observational studies with longer follow-up, more heterogeneous populations, and the effects of other ED constituents. Finally, adolescents and their parents should be educated on the adverse nutritional content and subsequent health effects of ED so that they can make informed decisions about consumption.

Despite some limited beneficial short-term effects, ED should be considered a significant public health problem that warrants attention. The growing evidence base demonstrating associations between ED consumption and negative health effects and public health challenges point to the need for close surveillance and assessment of this issue by researchers and policy makers.

## Author Contributions

LA-S and KV conceptualized the topic, researched and analyzed the background literature, and wrote the manuscript, including interpretations. CL and SR researched and analyzed the background literature and wrote portions of the manuscript, including interpretations. MT and JM provided substantial scholarly guidance on the conception of the topic, manuscript draft and interpretation, and revised the manuscript critically for intellectual content. All the authors approved the final version of the manuscript, ensured the accuracy and integrity of the work, and agreed to be accountable for all aspects of the work.

## Conflict of Interest Statement

The authors declare that the research was conducted in the absence of any commercial or financial relationships that could be construed as a potential conflict of interest. The reviewer, MS, declared a shared affiliation, with no collaboration, with the authors to the handling editor.

## References

[1] Heckman M Sherry K Mejia D Gonzalez E Energy drinks: an assessment of their market size, consumer demographics, ingredient profile, functionality, and regulations in the United States. Compr Rev Food Sci Food Saf (2010) 9(3):303–17. 10.1111/j.1541-4337.2010.00111.x 33467819

[2] Malinauskas BM Aeby VG Overton RF Carpenter-Aeby T Barber-Heidal K. A survey of energy drink consumption patterns among college students. Nutr J (2007) 6:35. 10.1186/1475-2891-6-35 17974021 PMC2206048

[3] Kit BK Fakhouri TH Park S Nielsen SJ Ogden CL Trends in sugar-sweetened beverage consumption among youth and adults in the United States: 1999–2010. Am J Clin Nutr (2013) 98:180–8. 10.3945/ajcn.112.057943 23676424 PMC10401613

[4] Wang YC Bleich SN Gortmaker SL Increasing caloric contribution from sugar-sweetened beverages and 100% fruit juices among US children and adolescents, 1988–2004. Pediatrics (2008) 121(6):e1604–14. 10.1542/peds.2007-2834 18519465

[5] Reissig CJ Strain EC Griffiths RR Caffeinated energy drinks – a growing problem. Drug Alcohol Depend (2009) 99(1–3):1–10. 10.1016/j.drugalcdep.2008.08.001 18809264 PMC2735818

[6] Clauson KA Shields KM McQueen CE Persad N. Safety issues associated with commercially available energy drinks. J Am Pharm Assoc (2003) (2008) 48(3):e55–63. 10.1331/JAPhA.2008.07055 18595815

[7] Breda JJ Whiting SH Encarnação R Norberg S Jones R Reinap M Energy drink consumption in Europe: a review of the risks, adverse health effects, and policy options to respond. Front Public Health (2014) 2:134. 10.3389/fpubh.2014.00134 25360435 PMC4197301

[8] Seifert SM Schaechter JL Hershorin ER Lipshultz SE Health effects of energy drinks on children, adolescents, and young adults. Pediatrics (2011) 127(3):511–28. 10.1542/peds.2009-3592 21321035 PMC3065144

[9] Schneider MB Benjamin HJ. Sports drinks and energy drinks for children and adolescents: are they appropriate? Pediatrics (2011) 127(6):1182–9. 10.1542/peds.2011-0965 21624882

[10] Generali JA Energy drinks: food, dietary supplement, or drug? Hosp Pharm (2013) 48(1):5 10.1310/hpj4801-5 24421413 PMC3839443

[11] McLellan TM Caldwell JA Lieberman HR A review of caffeine’s effects on cognitive, physical and occupational performance. Neurosci Biobehav Rev (2016) 71:294–312. 10.1016/j.neubiorev.2016.09.001 27612937

[12] Temple JL. Caffeine use in children: what we know, what we have left to learn, and why we should worry. Neurosci Biobehav Rev (2009) 33(6):793–806. 10.1016/j.neubiorev.2009.01.001 19428492 PMC2699625

[13] Higgins JP Tuttle TD Higgins CL Energy beverages: content and safety. Mayo Clin Proc (2010) 85(11):1033–41. 10.4065/mcp.2010.0381 21037046 PMC2966367

[14] World Health Organization. WHO Calls on Countries to Reduce Sugars Intake among Adults and Children. Geneva (2015). Available from: http://www.who.int/mediacentre/news/releases/2015/sugar-guideline/en/

[15] Finnegan D The health effects of stimulant drinks. Nutr Bull (2003) 28(2):147–55. 10.1046/j.1467-3010.2003.00345.x

[16] Scholey A Haskell C Neurocognitive effects of guaraná plant extract. Drugs Future (2008) 33(10):869 10.1358/dof.2008.33.10.1250977

[17] Lu J-M Yao Q Chen C. Ginseng compounds: an update on their molecular mechanisms and medical applications. Curr Vasc Pharmacol (2009) 7(3):293–302. 10.2174/157016109788340767 19601854 PMC2928028

[18] Yunusa I Ahmed IM Energy-drinks: composition and health benefits. Bayero J Pure Appl Sci (2011) 4(2):186–91. 10.4314/bajopas.v4i2.38

[19] Heck CI De Mejia EG. Yerba mate tea (*Ilex paraguariensis*): a comprehensive review on chemistry, health implications, and technological considerations. J Food Sci (2007) 72(9):R138–51. 10.1111/j.1750-3841.2007.00535.x 18034743

[20] Caine JJ Geracioti TD. Taurine, energy drinks, and neuroendocrine effects. Cleve Clin J Med (2016) 83(12):895–904. 10.3949/ccjm.83a.15050 27938518

[21] Alford C Cox H Wescott R. The effects of red bull energy drink on human performance and mood. Amino Acids (2001) 21(2):139–50. 10.1007/s007260170021 11665810

[22] Howard MA Marczinski CA. Acute effects of a glucose energy drink on behavioral control. Exp Clin Psychopharmacol (2010) 18(6):553–61. 10.1037/a0021740 21186930

[23] Roehrs T Roth T. Caffeine: sleep and daytime sleepiness. Sleep Med Rev (2008) 12(2):153–62. 10.1016/j.smrv.2007.07.004 17950009

[24] Abian-Vicen J Puente C Salinero JJ González-Millán C Areces F Muñoz G A caffeinated energy drink improves jump performance in adolescent basketball players. Amino Acids (2014) 46(5):1333–41. 10.1007/s00726-014-1702-6 24599611

[25] Forbes SC Candow DG Little JP Magnus C Chilibeck PD. Effect of red bull energy drink on repeated Wingate cycle performance and bench-press muscle endurance. Int J Sport Nutr Exerc Metab (2007) 17(5):433–44. 10.1123/ijsnem.17.5.433 18046053

[26] Gallo-Salazar C Areces F Abián-Vicén J Lara B Salinero JJ Gonzalez-Millán C Enhancing physical performance in elite junior tennis players with a caffeinated energy drink. Int J Sports Physiol Perform (2015) 10(3):305–10. 10.1123/ijspp.2014-0103 25158287

[27] Perez-Lopez A Salinero JJ Abian-Vicen J Valades D Lara B Hernandez C Caffeinated energy drinks improve volleyball performance in elite female players. Med Sci Sports Exerc (2015) 47(4):850–6. 10.1249/MSS.0000000000000455 25051390

[28] Souza DB Del Coso J Casonatto J Polito MD Acute effects of caffeine-containing energy drinks on physical performance: a systematic review and meta-analysis. Eur J Nutr (2016) 56:13–27. 10.1007/s00394-016-1331-9 27757591

[29] Emond JA Gilbert-Diamond D Tanski SE Sargent JD. Energy drink consumption and the risk of alcohol use disorder among a national sample of adolescents and young adults. J Pediatr (2014) 165(6):1194–200. 10.1016/j.jpeds.2014.08.050 25294603 PMC4252708

[30] Evren C Evren B. Energy-drink consumption and its relationship with substance use and sensation seeking among 10th grade students in Istanbul. Asian J Psychiatr (2015) 15:44–50. 10.1016/j.ajp.2015.05.001 26006774

[31] Larson N DeWolfe J Story M Neumark-Sztainer D. Adolescent consumption of sports and energy drinks: linkages to higher physical activity, unhealthy beverage patterns, cigarette smoking, and screen media use. J Nutr Educ Behav (2014) 46(3):181–7. 10.1016/j.jneb.2014.02.008 24809865 PMC4023868

[32] Terry-McElrath Y O'Malley P Johnston L. Energy drinks, soft drinks, and substance use among United States secondary school students. J Addict Med (2014) 8:6–13. 10.1097/01.ADM.0000435322.07020.53 24481080 PMC3910223

[33] Azagba S Langille D Asbridge M. An emerging adolescent health risk: caffeinated energy drink consumption patterns among high school students. Prev Med (2014) 62:54–9. 10.1016/j.ypmed.2014.01.019 24502849

[34] Barrense-Dias Y Berchtold A Akre C Surís JC. Consuming energy drinks at the age of 14 predicted legal and illegal substance use at 16. Acta Paediatr (2016) 105(11):1361–8. 10.1111/apa.13543 27513298

[35] Holubcikova J Kolarcik P Geckova AM Reijneveld SA van Dijk JP. Regular energy drink consumption is associated with the risk of health and behavioural problems in adolescents. Eur J Pediatr (2017) 176:599–605. 10.1007/s00431-017-2881-4 28229268

[36] Park S Lee Y Lee JH Association between energy drink intake, sleep, stress, and suicidality in Korean adolescents: energy drink use in isolation or in combination with junk food consumption. Nutr J (2016) 15(1):87 10.1186/s12937-016-0204-7 27737671 PMC5064784

[37] Richards G Smith AP. A review of energy drinks and mental health, with a focus on stress, anxiety, and depression. J Caffeine Res (2016) 6(2):49–63. 10.1089/jcr.2015.0033 27274415 PMC4892220

[38] García A Romero C Arroyave C Giraldo F Sánchez L Sánchez J Acute effects of energy drinks in medical students. Eur J Nutr (2016). 10.1007/s00394-016-1246-5 27312565

[39] Grasser EK Yepuri G Dulloo AG Montani J-P. Cardio-and cerebrovascular responses to the energy drink Red Bull in young adults: a randomized cross-over study. Eur J Nutr (2014) 53(7):1561–71. 10.1007/s00394-014-0661-8 24474552 PMC4175045

[40] Grasser EK Miles-Chan JL Charrière N Loonam CR Dulloo AG Montani J-P. Energy drinks and their impact on the cardiovascular system: potential mechanisms. Adv Nutr (2016) 7(5):950–60. 10.3945/an.116.012526 27633110 PMC5015039

[41] Shah SA Chu BW Lacey CS Riddock IC Lee M Dargush AE. Impact of acute energy drink consumption on blood pressure parameters: a meta-analysis. Ann Pharmacother (2016) 50(10):808–15. 10.1177/1060028016656433 27340146

[42] Seifert SM Seifert SA Schaechter JL Bronstein AC Benson BE Hershorin ER An analysis of energy-drink toxicity in the National Poison Data System. Clin Toxicol (2013) 51(7):566–74. 10.3109/15563650.2013.820310 23879181

[43] Gunja N Brown JA. Energy drinks: health risks and toxicity. Med J Aust (2012) 196(1):46–9. 10.5694/mja11.10838 22256934

[44] Malik VS Popkin BM Bray GA Després J-P Willett WC Hu FB Sugar-sweetened beverages and risk of metabolic syndrome and type 2 diabetes. Diabetes Care (2010) 33(11):2477–83. 10.2337/dc10-1079 20693348 PMC2963518

[45] Schulze MB Manson JE Ludwig DS Colditz GA Stampfer MJ Willett WC Sugar-sweetened beverages, weight gain, and incidence of type 2 diabetes in young and middle-aged women. JAMA (2004) 292(8):927–34. 10.1001/jama.292.8.927 15328324

[46] Vartanian LR Schwartz MB Brownell KD. Effects of soft drink consumption on nutrition and health: a systematic review and meta-analysis. Am J Public Health (2007) 97(4):667–75. 10.2105/AJPH.2005.083782 17329656 PMC1829363

[47] Hu FB Resolved: there is sufficient scientific evidence that decreasing sugar-sweetened beverage consumption will reduce the prevalence of obesity and obesity-related diseases. Obes Rev (2013) 14(8):606–19. 10.1111/obr.12040 23763695 PMC5325726

[48] Hu FB Malik VS. Sugar-sweetened beverages and risk of obesity and type 2 diabetes: epidemiologic evidence. Physiol Behav (2010) 100(1):47–54. 10.1016/j.physbeh.2010.01.036 20138901 PMC2862460

[49] Malik VS Schulze MB Hu FB. Intake of sugar-sweetened beverages and weight gain: a systematic review. Am J Clin Nutr (2006) 84(2):274–88. 16895873 10.1093/ajcn/84.1.274 PMC3210834

[50] DiMeglio DP Mattes RD. Liquid versus solid carbohydrate: effects on food intake and body weight. Int J Obes (2000) 24(6):794. 10.1038/sj.ijo.0801229 10878689

[51] Touger-Decker R Van Loveren C. Sugars and dental caries. Am J Clin Nutr (2003) 78(4):881S–92S. 14522753 10.1093/ajcn/78.4.881S

[52] Marshall TA Levy SM Broffitt B Warren JJ Eichenberger-Gilmore JM Burns TL Dental caries and beverage consumption in young children. Pediatrics (2003) 112(3):e184–91. 10.1542/peds.112.3.e184 12949310

[53] Marczinski CA Fillmore MT. Energy drinks mixed with alcohol: what are the risks? Nutr Rev (2014) 72(Suppl 1):98–107. 10.1111/nure.12127 25293549 PMC4190582

[54] Sanchez-Lozada LG Tapia E Jimenez A Bautista P Cristobal M Nepomuceno T Fructose-induced metabolic syndrome is associated with glomerular hypertension and renal microvascular damage in rats. Am J Physiol Renal Physiol (2007) 292(1):F423–9. 10.1152/ajprenal.00124.2006 16940562

[55] Gersch MS Mu W Cirillo P Reungjui S Zhang L Roncal C Fructose, but not dextrose, accelerates the progression of chronic kidney disease. Am J Physiol Renal Physiol (2007) 293(4):F1256–61. 10.1152/ajprenal.00181.2007 17670904

[56] Bashir D Reed-Schrader E Olympia RP Brady J Rivera R Serra T Clinical symptoms and adverse effects associated with energy drink consumption in adolescents. Pediatr Emerg Care (2016) 32(11):751–5. 10.1097/PEC.0000000000000703 27176902

[57] Koivusilta L Kuoppamäki H Rimpelä A. Energy drink consumption, health complaints and late bedtime among young adolescents. Int J Public Health (2016) 61(3):299–306. 10.1007/s00038-016-0797-9 26888471

[58] Kristjansson AL Sigfusdottir ID Mann MJ James JE Caffeinated sugar-sweetened beverages and common physical complaints in Icelandic children aged 10–12years. Prev Med (2014) 58:40–4. 10.1016/j.ypmed.2013.10.011 24494227

[59] Huhtinen H Lindfors P Rimpelä A Adolescents’ use of energy drinks and caffeine induced health complaints in Finland. Eur J Public Health (2013) 23(Suppl 1):ckt123.050 10.1093/eurpub/ckt123.050

[60] Natural Products Insider. Energy Drink Sales Will Skyrocket to $21 Billion. (2017). Available from: http://www.naturalproductsinsider.com/news/2013/02/energy-drink-sales-will-skyrocket-to-21-billion-b.aspx

[61] Dolan K The Soda with Buzz. Forbes. (2016). Available from: https://www.forbes.com/forbes/2005/0328/126.html

[62] United States Census Bureau. Population Estimates 2015. (2016). Available from: https://www.census.gov/topics/population.html

[63] Pechmann C Levine L Loughlin S Leslie F Impulsive and self-conscious: adolescents’ vulnerability to advertising and promotion. J Public Policy Mark (2005) 24(2):202–21. 10.1509/jppm.2005.24.2.202

[64] Markey EJ Durbin RJ Blumenthal R Buzz Kill: A Survey of Popular Energy Drinks Finds Majority of the Market Unwilling to Make Commitments to Protect Adolescents. (2016). Available from: http://www.markey.senate.gov/imo/media/doc/2014-12-30-Report_BuzzKill_EnergyDrinks_ScreenV.pdf

[65] Heckman MA Sherry K Mejia EGD Energy drinks: an assessment of their market size, consumer demographics, ingredient profile, functionality, and regulations in the United States. Compr Rev Food Sci Food Saf (2010) 9(3):303–17. 10.1111/j.1541-4337.2010.00111.x 33467819

[66] American Beverage Association. ABA Guidance for the Responsible Labeling and Marketing of Energy Drinks. (2014). Available from: http://www.ameribev.org/files/resources/2014-energy-drinks-guidance-approved-by-bod-43020.pdf

[67] Colchero MA Popkin BM Rivera JA Ng SW. Beverage purchases from stores in Mexico under the excise tax on sugar sweetened beverages: observational study. BMJ (2016) 352:h6704. 10.1136/bmj.h6704 26738745 PMC4986313

[68] Silver LD Ng SW Ryan-Ibarra S Taillie LS Induni M Miles DR Changes in prices, sales, consumer spending, and beverage consumption one year after a tax on sugar-sweetened beverages in Berkeley, California, US: a before-and-after study. PLoS Med (2017) 14(4):e1002283. 10.1371/journal.pmed.1002283 28419108 PMC5395172

[69] Zucconi S Volpato C Adinolfi F Gandini E Gentile E Loi A Gathering consumption data on specific consumer groups of energy drinks. EFSA Supporting Publications (2013) 10(3):1–190. 10.2903/sp.efsa.2013.EN-394

[70] Marczinski CA. Can energy drinks increase the desire for more alcohol? Adv Nutr (2015) 6(1):96–101. 10.3945/an.114.007393 25593148 PMC4288285

[71] Marczinski CA Fillmore MT Maloney SF Stamates AL Faster self-paced rate of drinking for alcohol mixed with energy drinks versus alcohol alone. Psychol Addict Behav (2017) 31(2):154 10.1037/adb0000229 27819431 PMC5344720

[72] Weldy DL. Risks of alcoholic energy drinks for youth. J Am Board Fam Med (2010) 23(4):555–8. 10.3122/jabfm.2010.04.090261 20616299

[73] Worland J Why You Might Not Want to Mix Alcohol and Energy Drinks. TIME (2015). Available from: http://time.com/3677044/alcohol-energy-drinks/

[74] Arria AM Caldeira KM Bugbee BA Vincent KB O’Grady KE Energy drink use patterns among young adults: associations with drunk driving. Alcohol Clin Exp Res (2016) 40(11):2456–66. 10.1111/acer.13229 27676240 PMC5074694

